# Determination and Pharmacokinetics of Di-(2-ethylhexyl) Phthalate in Rats by Ultra Performance Liquid Chromatography with Tandem Mass Spectrometry

**DOI:** 10.3390/molecules180911452

**Published:** 2013-09-16

**Authors:** Wan-Ling Chang-Liao, Mei-Ling Hou, Li-Wen Chang, Chia-Jung Lee, Yin-Meng Tsai, Lie-Chwen Lin, Tung-Hu Tsai

**Affiliations:** 1 Institute of Traditional Medicine, School of Medicine, National Yang-Ming University, No. 155, Sec. 2, Li-Nong St, Beitou District, Taipei 112, Taiwan; E-Mails: wling311@yahoo.com.tw (W.-L.C-L.); maylinghou@gmail.com (M.-L.H.); liwenchang104@gmail.com (L.-W.C.); m303092003@tmu.edu.tw (C.-J.L.); maguey1117@gmail.com (Y.-M.T.); lclin@nricm.edu.tw (L.-C.L.); 2 National Research Institute of Chinese Medicine, No. 155-1, Sec. 2, Li-Nong St., Beitou District, Taipei 11221, Taiwan; 3 Graduate Institute of Acupuncture Science, China Medical University, No. 91, Hsueh-Shih Road, Taichung 404, Taiwan; 4 Department of Education and Research, Taipei City Hospital, No.145, Zhengzhou Rd., Datong Dist., Taipei 103, Taiwan

**Keywords:** di-(2-ethylhexyl) phthalate (DEHP), fecal excretion, pharmacokinetics, phthalates, plasticizer

## Abstract

Di-(2-ethylhexyl) phthalate (DEHP) is used to increase the flexibility of plastics for industrial products. However, the illegal use of the plasticizer DEHP in food and drinks has been reported in Taiwan in 2011. In order to assess the exact extent of the absorption of DEHP via the oral route, the aim of this study is to develop a reliable and validated ultra performance liquid chromatography with tandem mass spectrometry (UPLC-MS/MS) method to evaluate the oral bioavailability of DEHP in rats. The optimal chromatographic separation of DEHP and butyl benzyl phthalate (BBP; used as internal standard) were achieved on a C_18_ column. The mobile phase was consisted of 5 mM ammonium acetate-methanol (11:89, v/v) with a flow rate of 0.25 mL/min. The monitoring ion transitions were *m/z* 391.4 → 149.0 for DEHP and *m/z* 313.3 → 149.0 for BBP. The mean matrix effects of DEHP at low, medium and high concentrations were 94.5 ± 5.7% and 100.1 ± 2.3% in plasma and feces homogenate samples, respectively. In conclusion, the validated UPLC-MS/MS method is suitable for analyzing the rat plasma sample of DEHP and the oral bioavailability of DEHP was about 7% in rats.

## 1. Introduction

DEHP has been widely used as a plasticizer in industry to improve the flexibility of polyvinylchloride. Although it is legal for DEHP to be used as an additive in plastic bottles, consumer products and food containers, previous studies have demonstrated that detectable levels of DEHP in food and medical devices, such as tubing, blood bags, and dialysis equipment, may pose potential health risks [1,2]. A variety of additives, including gum arabic, palm oil and emulsifiers, are commonly used in beverages as clouding agents to keep emulsions properly dispersed and enhance viscosity in conventional food products. However, in order to reduce cost, extend shelf life and give products a more appealing appearance, some manufacturers have added DEHP to clouding agents since the 1980s. In May of 2011, the Taiwanese Food and Drug Administration (TFDA) detected that DEHP had been used to replace palm oil as a clouding agent in food and drink.

DEHP has also been recognized as a kind of environmentally active hormone, and many of these toxic agents are associated with developmental and reproductive problems in laboratory animals and humans. In animal studies, DEHP caused a decrease in the ratio of estradiol to estrone levels and altered estradiol production and metabolism in female rats at a dose of 1,000 mg/kg [3]. Reproductive and developmental toxicity in sexual organs of male rats were also found in that study. It was also found that DEHP exposure during the prenatal period had adverse effects on the functions of liver, kidney and testes in male rats [4], while DEHP also altered reproductive performance by displaying antiandrogenic activity [5]. Exposure to high doses of DEHP in food stimulated hepatocyte proliferation and accelerated the development of hepatocellular carcinomas in both sexes of rats and mice [6,7].

Because of these toxicological and health concerns, the absorption and disposition of DEHP have been systematically investigated in different species. One preliminary study reported that ^14^C-labeled DEHP disappeared rapidly from the blood and approximately 57% of the total dose was recovered in the feces and 42% in the urine after intravenous administration to rats [8]. In the marmoset, bioavailability of DEHP was considered to be lower and the distribution of DEHP in the tissue was liver > kidney > testes following 2,000 mg/kg/day treatment for seven consecutive days [9]. Since the major route of exposure to DEHP for the general population is food and water intake [10], data concerning the amount of absorbed DEHP is an important index for evaluating the safety of oral exposure. In recent years, a number of techniques have been proposed for estimating the levels of DEHP in different matrixes. A NMR (nuclear magnetic resonance) spectroscopy study was performed to confirm the presence of DEHP [11]. Although HPLC-UV has been used for phthalate determination in environmental and biological samples, UV detection of DEHP is limited by low sensitivity and selectivity [12,13]. Gas chromatography (GC) with mass spectrometric detection offers some advantages for the quantification of DEHP in tissue extracts and plasma samples [14,15]. However, lipid material can significantly reduce the performance of GC–MS due to accumulation in the injection port, column and ionization source [16]. LC-MS/MS provides a rapid, selective and convenient method for phthalate analysis [17,18,19].

Based on this literature survey, our hypothesis is that DEHP will be rapidly absorbed and degraded through an enzymatic process [20,21]. To detect small amounts of analyte in the biological fluid and samples, a sensitive and reliable detected method is required. The present study develops a validated UPLC-MS/MS method using ultra-performance narrow bore column for the determination of oral bioavailability and elimination of DEHP in rats.

## 2. Results and Discussion

### 2.1. Optimization of UPLC-MS/MS Conditions

**Figure 1:**
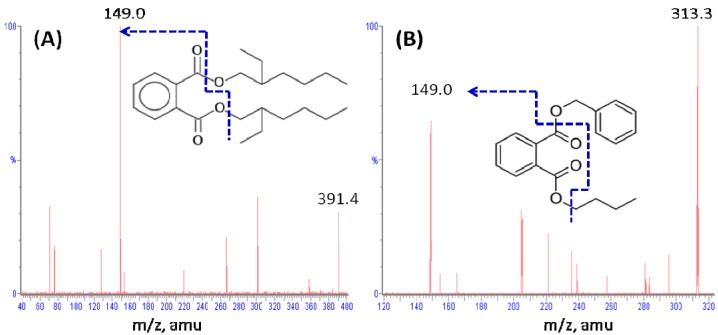
Representative product ion mass spectra and chemical structures of (**A**) DEHP and (**B**) BBP (internal standard).

**Figure 2:**
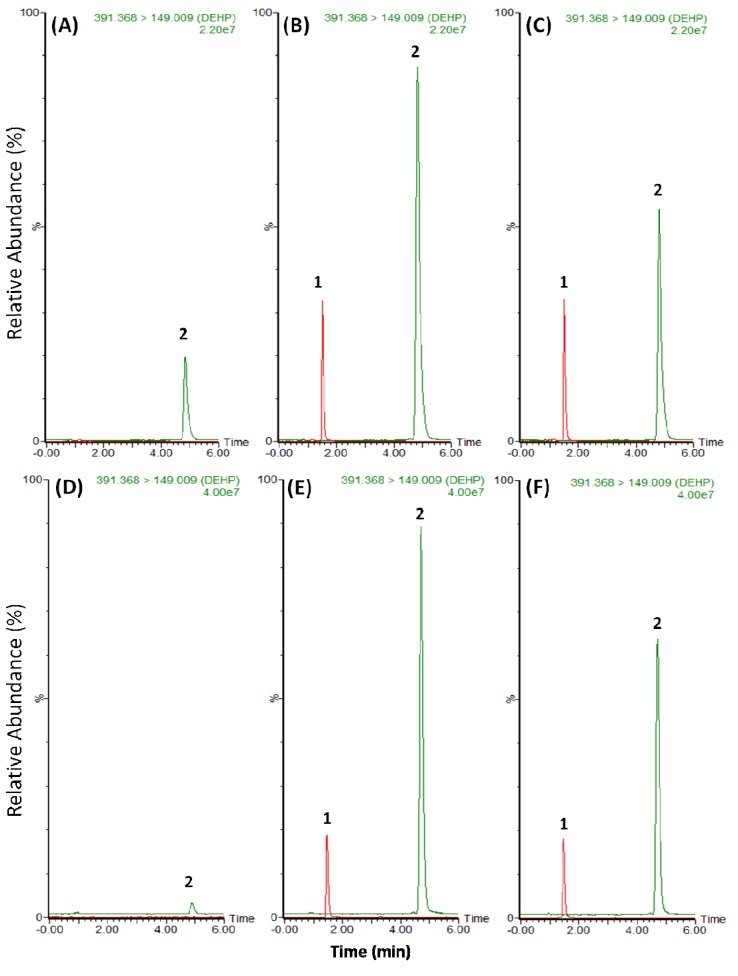
Representative UPLC-MS/MS chromatograms for the analytes. (**A**) Blank plasma sample; (**B**) blank plasma sample spiked with 0.5 μg/mL DEHP standard, and (**C**) plasma sample collected from rat plasma after administration of DEHP (10 mg/kg, i.v.). (**D**) Blank fecal sample; (**E**) blank fecal sample spiked with 1 μg/mL DEHP standard, and (**F**) fecal sample (400-fold dilution) collected from rat feces after administration of DEHP (100 mg/kg, p.o.) at 0–12 h. 1. Internal standard (BBP 1 μg/mL); 2. DEHP.

Positive ion electrospray ionization was used to optimize the UPLC-MS/MS analysis. Representative product ion mass spectra of DEHP and BBP are shown in Figure 1. The precursor ions of DEHP and BBP are located at *m/z* 391.4 [M+H]^+^ and *m/z* 313.3 [M+H]+, respectively, and the selected ions were fragmented in the collision cell. The most abundant transition product for multiple reaction monitoring (MRM) quantification of DEHP and BBP was *m/z* 149, corresponding to a phthalic anhydride fragment which can be derived from phthalate [21]. The Acquity UPLC BEH C18 column used in the study provided an effective separation of BBP and DEHP with retention times at 1.5 and 4.8 min, respectively. Typical UPLC-MS/MS chromatograms obtained after protein precipitation from rat plasma and fecal homogenates are shown in Figure 2. The elution conditions allowed for a complete runtime in less than 6 min, indicating that this high-throughput bioanalytical method for the analysis of DEHP was quick and suitable for the pharmacokinetic studies.

### 2.2. Background Contamination

Due to the ubiquitous background presence of DEHP in the general population, it was difficult to obtain a blank matrix from an animal source to use for the analysis of phthalates. However, the contamination level can be reduced by careful selection of analytical tools, glassware, solvents, and frequent verification of the chromatographic system [22,23]. In order to quantify DEHP contamination in blank samples, one previous study applied a column switching LC-MS coupled with an online sample preparation method [24]. In the present study, we simplified the sample preparation procedure by using protein precipitation with methanol to avoid any possibility of DEHP contamination. Furthermore, the calibration curve for each individual rat was established before DEHP administration in the study. Hence, the basal level of DEHP in blank sample had no influence on quantifying the DEHP in plasma and feces.

### 2.3. Method Validation

To assess the analytical range of the developed method, various concentrations of DEHP in blank sample ranging from 0.1 to 2.5 μg/mL were tested. The linear trends of standard curves were calculated by the standard addition method [25]. The results showed a good correlation coefficient (r^2^ ≥ 0.995) for DEHP over the concentration range from separately prepared analytical runs on different days. The limit of quantification (LOQ) was defined as the concentration of DEHP detected as signal-to-noise (S/N) ratio of 10.

As shown in Table 1, five replicates of each freshly prepared calibration standard were used for the determination of intra-day and inter-day accuracy (Bias %) and precision (R.S.D. %). Intra-day accuracy ranged from −1.0 to 3.1% with a precision ranged from 4.7 to 12.7%, while inter-day accuracy ranged from −3.3 to 6.7% with a precision ranged from 0.7% to 12.5% for plasma samples. For fecal samples, the intra-day and inter-day accuracy and precision of DEHP were within the validation criteria of the assay, which clarified that a 400-fold dilution would be acceptable for fecal samples. These data showed that all replicate measurements were accurate, precise and reproducible for the quantification of DEHP in biological samples.

**Table 1:** Accuracy and precision of DEHP in rat plasma and feces.

Nominal concentration (μg/mL)	Bias (%)	R.S.D. (%)		Bias (%)	R.S.D. (%)
	plasma			feces	
**Intra-day**					
0.1	3.1%	7.4%		−6.9%	8.6%
0.25	−1.0%	12.7%		4.6%	5.5%
0.5	1.9%	4.7%		7.3%	4.1%
1	−0.5%	4.7%		−1.3%	4.0%
2.5	1.0%	5.2%		0.1%	0.6%
**Inter-day**					
0.1	6.7%	12.5%		6.5%	17.5%
0.25	3.1%	8.1%		5.5%	7.5%
0.5	1.0%	7.4%		−5.2%	9.3%
1	−3.3%	5.1%		−0.2%	4.1%
2.5	0.4%	0.7%		0.2%	0.9%

Matrix effect results from co-eluting matrix components can influence ionization of the target analyte, resulting in either ion suppression or ion enhancement [26]. Although protein precipitation failed to completely remove the unwanted plasma components, the matrix effect of DEHP for both plasma and feces in the methanol extract ranged from 88.5 to 99.8% and 98.3 to 102.7%, which were both considered acceptable (Table 2). Compared with the neat solution, a higher matrix effect of the internal standard was observed in the plasma sample, while there was no significant matrix effect in feces. Since the value of the matrix effect in plasma had only a small standard deviation, the BBP can be used as a suitable internal standard for the analysis. Our results also showed the recovery values of DEHP were between 87.4% and 104.8% with standard deviation less than 5.9% in biological samples (Table 2).

**Table 2:** Matrix effect and recovery of DEHP and BBP (internal standard, IS) in rat plasma and feces after sample preparation.

Nominal concentration (μg/mL)		Peak area			Matrix effect (%)	Recovery (%)
		Set 1	Set 2	Set 3		
plasma DEHP	0.1	404927 ± 8274	403570 ± 25876	352369 ± 11799	99.8 ± 8.4	87.4 ± 2.9
	0.5	2031609 ± 188125	1923814 ± 37163	1773849 ± 51976	95.3 ± 9.9	92.2 ± 1.3
	2.5	11178107 ± 157242	9886720 ± 523536	8874023 ± 176644	88.5 ± 5.0	89.9 ± 4.7
Mean ± SD					94.5 ± 5.7	89.8 ± 2.4
BBP (IS)	1	141988 ± 3666	92634 ± 1572	92775 ± 2371	65.3 ± 2.7	100.2 ± 3.4
feces DEHP	0.1	430319 ± 18291	442401 ± 42730	453572 ± 19591	102.7 ± 7.2	102.9 ± 5.9
	0.25	957870 ± 5154	942234 ± 52151	941791 ± 12950	98.4 ± 5.7	100.1 ± 5.3
	1	3661538 ± 100557	3625061 ± 87726	3798477 ± 194145	99.1 ± 4.6	104.8 ± 4.0
Mean ± SD					100.1 ± 2.3	102.6 ± 2.4
BBP (IS)	1	134186 ± 13012	131132 ± 3432	131577 ± 3232	98.3 ± 9.3	100.4 ± 3.2

Data are presented as means ± S.D. (n = 3). Matrix effect (%) = (SET 2/SET 1) × 100; Recovery (%) = (SET 3/SET 2) × 100; SET 1: the peak areas obtained in neat solution standards; SET 2: the peak areas for standards spiked after extraction; SET 3: the peak areas for standards spiked before extraction.

### 2.4. Pharmacokinetic Studies of DEHP Administration

**Figure 3:**
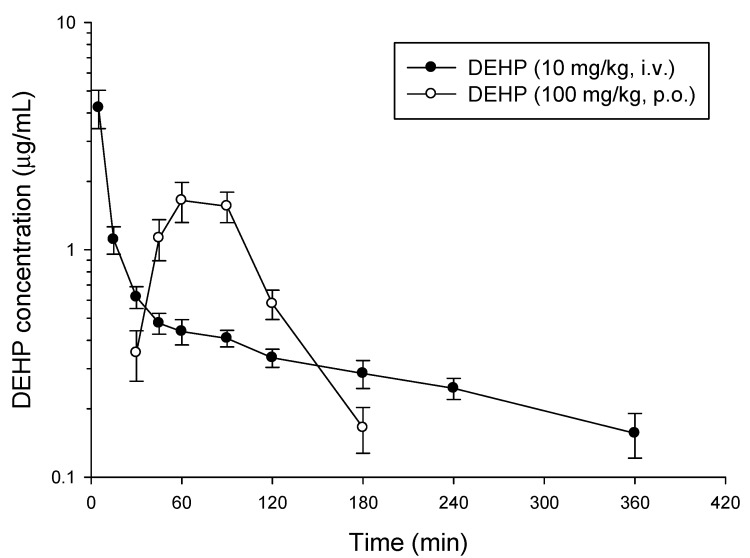
Concentration-time profile of DEHP after drug administration (10 mg/kg, i.v.; 100 mg/kg, p.o.) in rat plasma. Data are presented as mean ± S.E.M. (n = 6).

The basic pharmacokinetic and bioavailability information on DEHP were obtained after a single intravenous and oral administration to rats at the doses of 10 and 100 mg/kg, respectively. The mean concentration versus time profiles of DEHP with a single intravenous and oral administration to six individual rats are depicted in Figure 3, and the pharmacokinetic parameters are listed in Table 3. In the intravenous group, DEHP achieved a C_max_ of 4.2 ± 0.8 μg/mL, an AUC of 220 ± 23 min μg/mL, an elimination half-life of 192 ± 33 min and a volume of distribution of 12.5 ± 1.6 L/kg. The time courses of DEHP concentrations in plasma after the intravenous application showed up over 6 h, whereas DEHP was rapidly eliminated after oral administration. According to our results, DEHP reached a C_max_ within approximately 60–90 min with a value of 1.8 ± 0.3 μg/mL after oral dosing. The T_max_ of DEHP after oral administration was 75 ± 6.71 min. The elimination half-life of DEHP was 31 ± 5 min and the volume of distribution was 34.9 ± 8.4 L/kg. The oral bioavailability of DEHP calculated by the equation described above was about 6.74 ± 0.92%.

**Table 3:** Pharmacokinetic data of DEHP in rat plasma.

Pharmacokinetic parameters	DEHP (10 mg/kg, i.v.)	DEHP (100 mg/kg, p.o.)
C_max_ (μg/mL)	4.2 ± 0.8	1.8 ± 0.3
AUC (min μg/mL)	220 ± 23	148 ± 20
T_max_ (min)		75 ± 6.71
t_1/2_ (min)	192 ± 33	31 ± 5
Cl (mL/min per kg)	49.6 ± 8.0	751 ± 119
MRT (min)	223 ± 55	94 ± 7
Vd (L/kg)	12.5 ± 1.6	34.9 ± 8.4
Bioavailability (%)		6.74 ± 0.92

The disposition kinetics of DEHP and its mono-de-esterified metabolite in rats has been reported [27]. Pollack *et al.* [27] have investigated that the systemic availability of DEHP was low following both a single oral (13.6%) and an intraperitoneal (5.2%) administration. In addition, the kinetics of intestinal absorption of DEHP has been estimated indirectly by measuring extractable ^14^C-labeled radioactivity from the blood [8]. It has been proposed that the conversion of DEHP to MEHP occurs almost exclusively before absorption at low doses, whereas at high doses, some intact DEHP may be absorbed as well [28,29]. Since DEHP is rapidly metabolized in the gut after oral exposure, the amount of ^14^C-DEHP radioactivity was negligible in male CFN rat tissues, including spleen, lung, kidney, heart, fat, thymus, brain and testes after 24 h following a single 0.1 mg/kg dose of DEHP [30]. However, the exposure level of DEHP at 200 mg/kg lead to continuously measured of radioactivity in the liver, spleen, lung, and stomach for 24 h [30].

Given single infusions of a DEHP emulsion in doses of 5, 50 or 500 mg/kg to rats, nonlinear increases in the AUCs of DEHP and MEHP indicated saturation in the formation as well as the elimination of the potentially toxic metabolite MEHP [31]. Testosterone production and gene endpoints in the fetal testis have been considered as the critical effect in some phthalate risk assessments, and thus the dose-response relationship for the effects of DEHP on the synthesis and production of testosterone in the fetal rat testis has been investigated [32]. The results indicated that alteration in testosterone production and in key steroidogenic gene expression were apparent at lower doses than those causing postnatal reproductive malformations after gestational exposure during the critical period of male sexual differentiation [32].

The predominant exposure pathways to DEHP for the general population are ingested food and water [10]. The Food and Drug Administration has suggested that the extrapolation of animal dose to human dose is correctly performed only through normalization to body surface area (BSA), which often is represented in mg/m^2^. The human dose equivalent can be more appropriately calculated by using the formula, human equivalent dose (HED, mg/kg) = Animal dose (mg/kg) multiplied by (animal Km/human Km) [33]. For more appropriate conversion of DEHP doses from animal studies to human studies, the body surface area normalization method was employed [33]. Based on chronic toxicity animal studies, there were no treatment-related changes in organ (kidneys, liver, lungs, testes, uterus, brain, and spleen) weights of the Fischer 344 rats treated with DEHP at 100 and 500 ppm in the diet for up to 104 weeks; however, rats treated with DEHP at 12,500 ppm in the diet for 104 weeks, the mean liver weights were significantly increased [34]. According to the European Union risk assessment report, typical human exposure of total phthalate esters was estimated to be 0.3 mg/kg body weight [35]. Previous research has proposed the average total daily exposure to DEHP is about 0.21–2.1 mg/day in an adult [36,37,38]. Based on our findings, the oral bioavailability of DEHP at dose of 100 mg/kg in rat was 7%; it is indicated that 7 mg/kg which is equivalent to the DEHP administration dose of 1.13 mg/kg in human was absorbed into the rat. However, the proposed daily exposure to DEHP is about 0.21–2.1 mg/kg in an adult according to the previous study [36,37,38] so that even short term exposures to the low doses of DEHP in daily life from food may be considered a potential risk factor.

### 2.5. Fecal Excretion Profiles of DEHP

**Figure 4:**
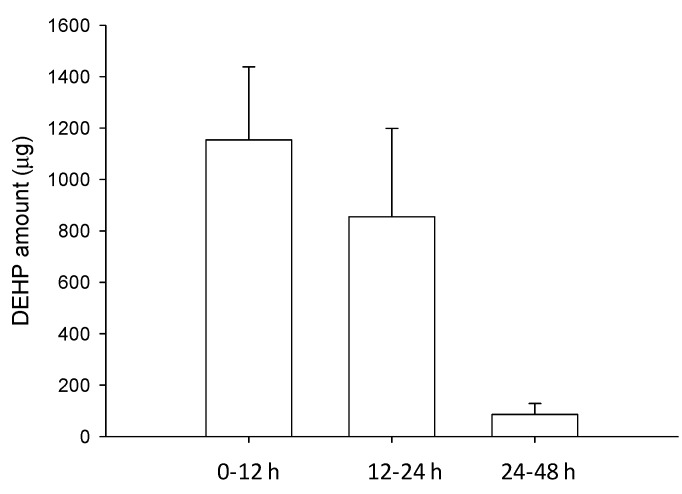
Fecal excretion profile of DEHP following a single oral administration of DEHP (100 mg/kg); results are expressed as mean ± S.E.M. (n = 6).

To determine the extent of DEHP uptake, the total amount of DEHP excreted or lost through the urine and feces must be considered. In the present study, we presented DEHP levels in rat feces after a single oral ingestion of DEHP over different time intervals (Figure 4). Approximately 10.64% of administered dose was recovered in feces within 48 h, although DEHP could not be detected in the urine (data not shown); illustrating that there was a low fecal excretion of DEHP in prototype. Previous studies have revealed that the level of parent phthalates was very low in the urine because of its fast metabolism to the monoester and other secondary metabolites [39,40]. The results of Teirlynck and Belpaire [41] demonstrated that when ^14^C-DEHP was administered to rats, 19.3% of the radioactivity was excreted in the urine within 72 h, the rest being excreted in the feces. Furthermore, human metabolism researches have demonstrated that the range of urinary excretion rates is from 10 to 31% after oral DEHP administration, and the metabolites pattern exhibits high ratio of the ω-oxidized metabolites, mono(2-ethyl-5-carboxypentyl)phthalate (5cx-MEPP) and mono [2-(carboxymethyl) hexyl] phthalate (2cx-MMHP), in the general population [42,43]. Koch *et al.* [43] have reported that most of the orally administered DEHP is systemically absorbed and excreted in urine. However, the metabolites of DEHP were not investigated in our study. Based on the published studies and our findings, and thus it is estimated that around 20% (6.74% bioavailability and 10.64% fecal excretion) of the dosage given DEHP could be detected as the original form.

## 3. Experimental

### 3.1. Chemicals and reagents

DEHP (CAS number: 117-81-7), ammonium acetate, acetonitrile and methanol were of analytical grade purchased from E. Merck (Darmstadt, Germany). Benzyl butyl phthalate (BBP) was obtained from Supelco (Bellefonte, PA, USA) and polyethylene glycol 400 (PEG 400) was obtained from Fluka AG (Buchs, Switzerland). Olive oil was supplied by Sigma-Aldrich (St. Louis, MO, USA). All aqueous solutions were prepared by triply deionized water from Millipore (Bedford, MA, USA) in the study.

### 3.2. Instrumental Analysis

The present study used a Waters Acquity UPLC™ system coupled to Waters XevoTM tandem quadruple mass spectrometer fitted with electrospray ionization probe (Waters Corporation, Milford, MA, USA). The chromatography was performed on an Acquity UPLC BEH C_18_ (2.1 mm × 100 mm, 1.7 μm) column (Waters Corporation) and maintained at 40 °C. The mobile phase was consisted of 5 mM ammonium acetate-methanol (11:89, v/v), and the flow rate was set at 0.25 mL/min. The injection volume was 5 µL. The positive ion mode with multiple reactions monitor (MRM) was used for UPLC-MS/MS analysis. The following conditions for monitoring DEHP were optimized: capillary voltage, 2.9 KV; ion source temperature, 150 °C; desolvation gas temperature, 400 °C; desolvation gas flow rate, 800 L/h. The cone voltage of 20 V and collision energy of 18 eV were deemed optimal for DEHP; the cone voltage of 34 V and collision energy of 10 eV were deemed optimal for BBP. Micromass MassLynx 4.1 software was used for data processing.

### 3.3. Method Validation

Method validation was performed based on the US FDA guidelines for bioanalytical method validation [44]. Stock standard solutions of DEHP were prepared in methanol and stored at −20 °C in Teflon-capped glass vials. A working standard solution was prepared by appropriate dilution of the stock solution with 50% methanol. Analytical characteristics of the method with respect to linearity, precision and accuracy were determined. The linearity of detector response was studied by using calibration solutions at five concentration levels, ranging from 0.1 to 2.5 μg/mL. The calibration curve was linear over the range of 0.1 to 2.5 μg/mL with the correlation coefficient (r^2^) greater than 0.995. The accuracy (bias %) was calculated from the mean value of observed concentration (C_obs_) and nominal concentration (C_nom_) using the relationship accuracy (bias %) = [(C_obs_ − C_nom_)/C_nom_] × 100. The relative standard deviation (RSD) was calculated from the observed concentrations as precision (RSD %) = [standard deviation (SD)/C_obs_] × 100. Intra-day and inter-day precision and accuracy of the method in rat plasma and feces were less than ± 15% (± 20% at the low limit of detection) for all analytes. Samples with concentrations higher than the highest standard in the calibration curve were diluted to appropriate DEHP concentrations.

The recovery and matrix effect of DEHP in an assay were obtained for the three replicates at three different concentration levels. The recovery from the extraction was obtained by comparing the peak areas of analytes spiked into pre-extracted samples with analytes spiked into post-extracted samples. The matrix effect was calculated by comparing the mean peak areas of analytes spiked after extraction to the values of analytes dissolved in matrix-free solvent.

### 3.4. Experimental Animals

Male Sprague-Dawley rats (210 ± 20 g) were obtained from the Laboratory Animal Center at National Yang-Ming University (Taipei, Taiwan) and were housed at constant room temperature with a 12-hour light/dark cycle and free access to food (Laboratory Rodent Diet 5001, PMI Feeds Inc., Richmond, IN, USA) and water. All experimental protocols involving animals were reviewed and approved by the Institutional Animal Care and Use Committee (IACUC number: 1000802) of National Yang-Ming University and were carried out in accordance with the Guide for the Care and Use of Laboratory Animals of the National Institutes of Health.

Six individual Sprague-Dawley rats were used for each pharmacokinetic study. To evaluate the pharmacokinetics of DEHP, rats were anesthetized intraperitoneally with pentobarbital sodium (50 mg/kg). Body temperature was maintained by using a heating pad placed underneath the rats during surgery. A polyethylene tube (PE-50) was implanted into the femoral vein for administering the DEHP and the right jugular vein for collecting blood. For the oral administration group (n = 6), only the right jugular vein was catheterized for blood sampling. Patency of the tube was maintained by flushing with heparinized saline (20 IU/mL). Rats were allowed to recover 24 h prior to drug administration. For intravenous administration (n = 6), DEHP was dissolved in PEG 400 and given via the femoral vein at the dose of 10 mg/kg. In the oral group, DEHP was suspended in olive oil and administered by gastric gavage at the dose of 100 mg/kg. Repeated sampling of blood and continuous collection of urine and feces were acquired for the freely behaving, unrestrained rats placed in metabolism cages (Mini Mitter, Bend, OR, USA) for a period of 48 h. Blood samples (300 μL) for analysis of DEHP were withdrawn from the jugular vein cannula at 0, 5, 15, 30, 45, 60, 90, 120, 180, 240 and 360 min after dose administration. Plasma was separated by centrifugation at 6,000 × *g* for 10 min at 4 °C. The fecal and urinary samples were individually obtained from each animal at 0–12, 12–24, 24–48 h after DEHP oral treatment. The resulting biological sample was stored at −20 °C before analysis.

### 3.5. Sample Preparation

A volume of 50 μL rat plasma was mixed with 5 μL of internal standard solution (BBP, 10 μg/mL) and the solution was vortex-mixed with 145 μL methanol for protein precipitation. The protein precipitate was separated by centrifugation at 16,000 *g* for 10 min at 4 °C. Fecal samples were air-dried in the hood prior to weighing and were extracted with acetonitrile at a solvent-to-sample ratio of 3:1. A volume of 50 μL fecal supernatant was spiked with 5 μL of internal standard (BBP, 10 μg/mL) and 145 μL of methanol was added for protein precipitation followed by centrifugation at 16,000 × *g* for 10 min at 4 °C. The supernatant was diluted to the appropriate concentration with mobile phase. Then, the concentrations of DEHP in plasma and in feces were determined by UPLC-MS/MS as described above.

### 3.6. Pharmacokinetic Data Analysis

The pharmacokinetic models (one- or two-compartment) were compared according to the Akaike’s Information Criterion (AIC) [45] with minimum AIC values being regarded as the best representation for the plasma concentration-time course data. A compartment model with individual animal data after dose was proposed by the computer program WinNonlin. In the present study, concentration-time data for DEHP were analyzed using a non-compartmental model. The AUC_0-t_, the elimination half-life (t_1/2_), the clearance (Cl), the mean residence time (MRT) and the apparent volume of distribution (V_d_) were calculated. The software package used for this study was WinNonlin Standard Edition Version 1.1 (Scientific Consulting Inc., Apex, NC, USA). The calculation of bioavailability of DEHP was shown by the equation of bioavailability (%) = [(AUCp.o./Dose p.o.)/(AUCi.v./Dose i.v.) × 100%]. The pharmacokinetic results are presented as mean ± standard error mean (S.E.M.).

## 4. Conclusions

To our knowledge, this is the first study to directly evaluate the pharmacokinetics of DEHP in rats. We developed a fast and robust UPLC-MS/MS analytical method for the quantification of DEHP in the biological samples. The effective separation of BBP and DEHP in 6 min shows that this validated method is a high-throughput approach for analysis. The pharmacokinetic results were determined after intravenous and oral administration, demonstrating DEHP was rapidly eliminated from rats. Furthermore, feces and urine were minor routes of excretion of DEHP in rats. These findings provide the basis for further investigation of the possible risk assessment for DEHP due to oral exposure.
